# Optoionics: New opportunity for ionic conduction-based radiation detection

**DOI:** 10.1557/s43579-025-00726-9

**Published:** 2025-05-13

**Authors:** Thomas Defferriere, Harry L. Tuller

**Affiliations:** Department of Material Science and Engineering, Massachusetts Institute of Technology, Cambridge, MA 02139 USA

**Keywords:** Sensor, Radiation effects, Ceramics, Ionic conductor, Grain boundaries

## Abstract

Optoionics, involving light-modulated ionic transport in ionic solids, parallels optoelectronics in semiconductors and offers novel device design opportunities across various fields. Among these opportunities, grain boundary phenomena related to radiation-induced electron/hole pair generation and charge trapping at the boundaries causing a modulation in ionic current could enable fast, sensitive, and reversible radiation detectors. The robustness of ionic solids in chemical, structural, and thermal aspects in turn makes them scalable and robust alternatives to traditional semiconductor detectors. This article explores the theoretical underpinnings, experimental breakthroughs, and design considerations needed to optimize such optoionic devices.

**Graphical abstract:**

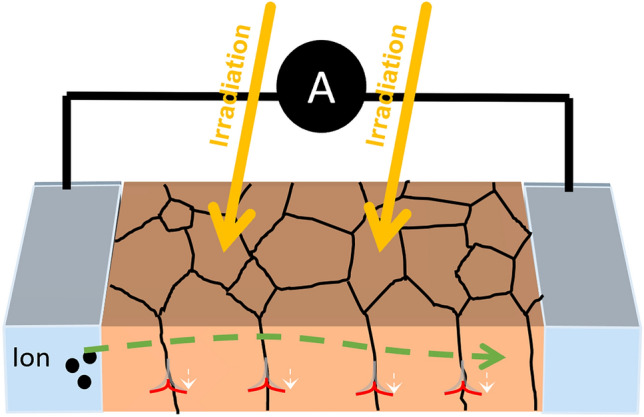

## Introduction

Radiation detection is a cornerstone technology in applications, such as nuclear power generation, medical diagnostics, environmental monitoring, and homeland security. The accurate and reliable detection of ionizing radiation, including gamma rays, neutrons, and x-rays, is essential for ensuring safety, enabling diagnostics, and advancing research. However, the performance of existing radiation detection technologies is constrained by fundamental material and operational challenges, particularly in extreme environments, such as high temperatures, corrosive atmospheres, and high-radiation fields, where the stability of conventional radiation detection materials is often compromised.^[1]^ In medical imaging, scaling detectors for large-area applications and improving sensitivity to reduce patient radiation exposure remain challenging due to technical and cost limitations.^[2,3]^ State-of-the-art high-performance radiation detectors are often based on semiconducting materials like Si, CdZnTe, or high-purity Ge in which radiation generated electron–hole pairs are accelerated under high DC fields and collected at the electrodes, with the current collected as a measure of radiation intensity. Charge counting schemes based on pulsed processing, in turn, allow for the determination of the energy spectra of the radiation, enabling isotopic identification. While such detectors offer high sensitivity and resolution, they are constrained by high fabrication costs and operational challenges. For instance, CdZnTe requires high-quality, defect-free crystals to limit electron–hole recombination and ensure high mobilities. These unfortunately are inherently expensive to grow and difficult to scale. While single-crystalline Ge detectors are commercially more feasible, they require cryogenic cooling to minimize noise from thermally excited carriers, owing to Ge’s low band gap (*E*_g_ ~ 0.6 eV). Even wider band gap semiconductors, such as SiC, which have been shown to exhibit superior performance under harsh environments, are still challenged by manufacturing constraints.^[4]^ Emerging materials, such as halide compounds (e.g., CsPbBr_3_, TlBr), promise lower costs and high performance, but can suffer from chemical instability, susceptibility to environmental degradation, and operational challenges like ionic defect migration and electrochemical reactions at the electrodes, altering the charge transfer process that generates the signal. These issues can complicate large-scale deployment and limit their use under harsh operating conditions (e.g., high temperatures, high fields, and/or corrosive chemical environments).

A fundamental shift in the design and operation of solid-state radiation detectors may allow for these limitations to be addressed. As we show in the following, polycrystalline metal oxide-based ionic conductors are promising alternative candidate materials due to their unique physical and chemical properties. This may at first be surprising given that these materials are characterized by high defect concentrations that enhance electron–hole recombination, combined with ionic charge carriers that exhibit many orders of magnitude lower mobilities than the electronic carrier counterparts in optimized semiconducting materials. However, these typically wide band gap materials (> 3 eV) are highly defect tolerant (~ 10^21^ atoms/cm^3^), while remaining structurally stable and chemically robust over broad temperature ranges. For example, oxygen and proton conducting solid electrolytes such as acceptor doped (trivalent cations at 10–20 at% substitution levels) of ZrO_2_, CeO_2_, LaGaO_3_, BaZrO_3_ or BaCeO_3_ are typically used in high temperatures fuel and electrolysis cells operated at temperatures > 600℃ for 1000 h’s under large chemical potential gradients (e.g., O_2_/Electrolyte/H_2_:H_2_O).^[5]^

While these materials are rarely considered for radiation detection purposes due to poor expected optoelectronic response (i.e., very short carrier lifetimes and mobilities), recent studies have shown that above-band gap irradiation can lead to modifications in ionic transport properties through a variety of mechanisms. Point defect generation or insertion associated with charge compensation of photogenerated charge carriers is one prime example. For example, Kim et al.^[6,7]^ reported that above-band gap illumination of the perovskite solar cell material (methylammonium)PbI_3_ (MAPI) led to the formation of mobile iodide vacancies, *via* hole trapping at iodine ions. This allows the resultant smaller neutral iodine atoms to insert themself interstitially into the lattice, creating iodine vacancy-interstitial pairs, thereby leading to both enhanced electronic (non-trapped electrons) and ionic conduction (iodine vacancies) over that in the dark. Upon removal of illumination, this process reverses, given recombination of the photogenerated electrons and holes and subsequent return of the iodide ions to their lattice sites.

While the optoionic effect discovered in MAPI is demonstrated and interesting, it has so far been limited to that specific material system owing to the unique nature of the polarizable iodide ions.^[8]^ Alternative optoionic processes have emerged that may be more broadly applicable. “Photo-intercalation,” predicted by Tributsch^[9]^ in 1980’s, that relies on photogenerated charge carriers to drive intercalation of ionic species into a solid from an external media is one example^[10]^ and has been demonstrated in several material systems.^[10–14]^ In all cases examined, however, light modifies both ionic and electronic conductivities, leading to difficulties in isolating the impact of illumination on the ionic component of the conductivity.^[6]^ Furthermore, the requirement that ionic species be intercalated between the material and external media limits response time and/or device configuration, thereby markedly restricting potential applicability as solid-state radiation detectors.

**Figure 1:**
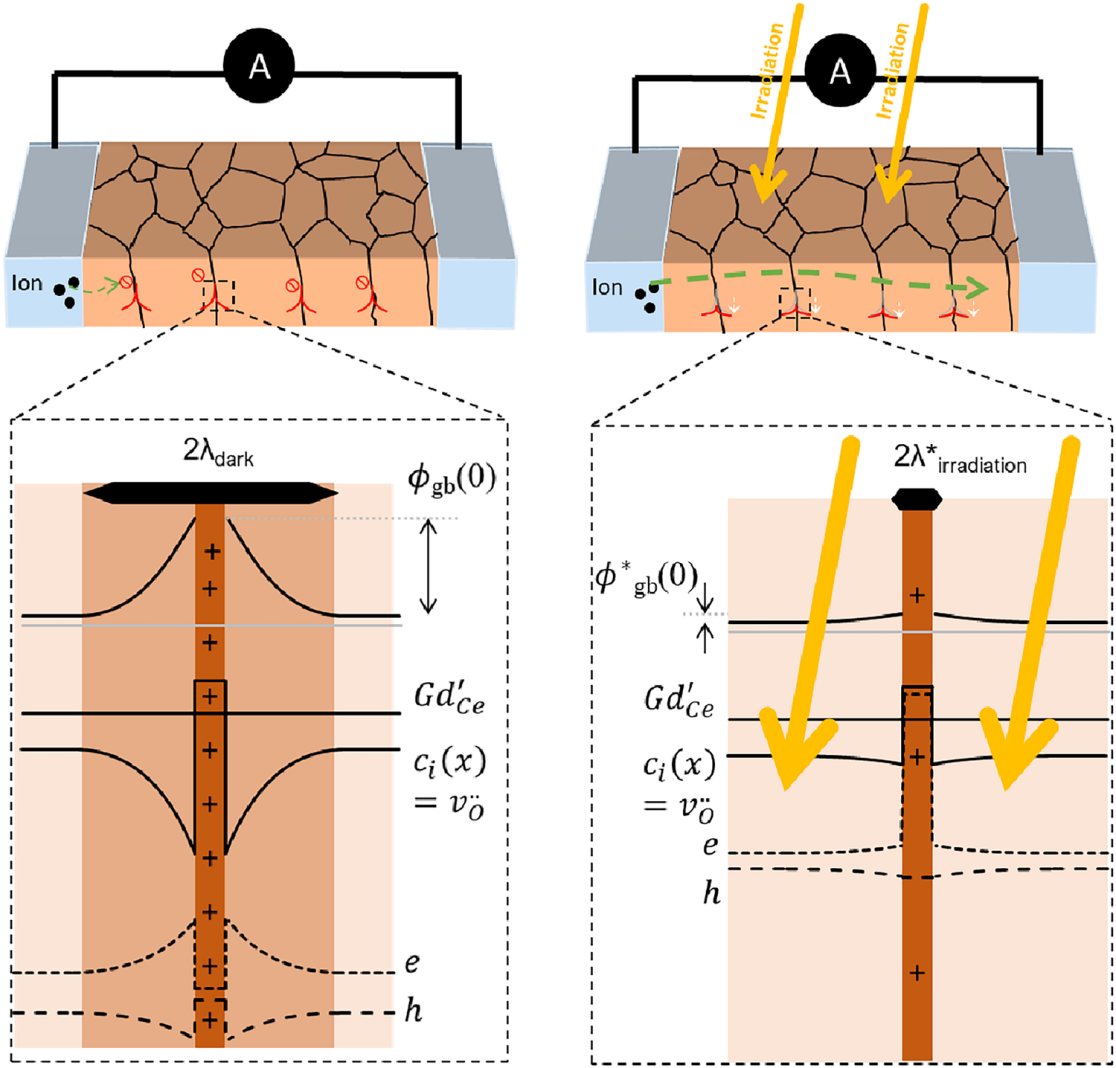
Schematic representation of oxygen ion-based radiation detection device. Top representations show grain boundaries acting as barriers to the transport of ions in the dark (top left), preventing the flow of current. Under above-band gap irradiation (top right), the potential barriers at the grain boundaries are reduced, allowing for an increased flow of ions. Bottom representation shows how potential barriers lead to modulation of defect concentrations in proximity of the interface. In the dark (bottom left), the majority defect concentrations (example: oxygen vacancies) are depleted by orders of magnitude in proximity to the interface. Upon above-band gap irradiation (bottom right), the barrier is reduced, leading to a reduction in defect depletion, and correspondingly an increase in ionic current in the device.

Recently, we demonstrated that in ion-conducting polycrystalline solid electrolytes, the dominant grain boundary resistance could be modulated by above-band gap illumination *via* modulation of the grain boundary (GB) space charge potential.^[15]^ This was first demonstrated in polycrystalline thin films of Gd-doped CeO_2_ (GDC) under ultraviolet (UV) illumination and was subsequently extended to bulk ceramic pellets of GDC under gamma irradiation (Co^60^ ~ 1 MeV).^[16]^ Because this phenomenon occurs in solid electrolytes where the defect concentration is fixed by extrinsic doping, these materials can exhibit remarkable structural stability, even under extended and high-dose irradiation, while demonstrating large reversible changes in resistance.^[16]^ In contrast to the other optoionic mechanisms described above, this GB mechanism does not result in a change in the total point defect concentrations but depends only on the local redistribution of mobile ions near the grain boundaries. This is in response to lowering electrostatic potential barriers and trapping of photogenerated charge carriers at the GB cores—as shown in Fig. 1. Given the short photogenerated electronic charge transport lengths toward the GBs (typically < 1 $\mu m$ grain size) and the efficient e^−^–h^+^ charge separation driven by the grain boundary space charge fields, this results in highly efficient localized signal generation. Since the grain boundaries trap the photogenerated charge, the GBs can therefore be thought of as “virtual electrodes” (in analogy to normal electrodes used to collect photogenerated charge carriers in semiconductor detectors). This can enable both fast response times and small interaction volumes with high collection efficiency. This optoionic effect enables a reversible and robust resistance change, opening the door to radiation detection systems that are cost-effective, scalable, and operable under extreme conditions. Moreover, this offers opportunities for designing detectors that do not require high electric fields, as the photogenerated charge carriers do not rely on external fields to separate the charges. This allows for processing in more conventional polycrystalline rather than single crystal forms, leading to wider area scalability and lower cost. Moreover, space charge phenomena at grain boundaries are universal in solid electrolytes. Such phenomena are therefore expected to be generally applicable to all intentionally engineered electrolyte systems, opening the door to the use of a broad range of both anionic (e.g., O^2−^, H^+^), and cationic (e.g., Li^+^, Na^+^, Ag^+^) species, each offering differences in response rate, stability, and ability to interact with a wide range of ionizing radiations that differ in their interactions with atomic nuclei (e.g., neutrons vs gamma rays), all the while taking advantage of the unique structural robustness of these ceramic materials. In this perspective, we aim to describe the fundamental physics underpinning the grain boundary optoionic effect. We aim to relate these mechanisms to device performance metrics, such as sensitivity, dynamic range, and response time. By elucidating these relationships, we aim to establish a foundation for designing and developing next-generation radiation detectors that are highly sensitive, robust, scalable, and environmentally adaptable.

## Fundamentals of ionic conduction

### Bulk ionic transport

#### Point defects

Ionic conduction in solids occurs through the thermally activated hopping of ions, facilitated by the presence of charged defects, such as vacancies or interstitials. The concentration of carriers is either intrinsically introduced due to inherent defect formation processes such as Schottky or Frenkel defect reactions or *via* equilibration with the environment *via* changes in stoichiometry. For example, undoped metal oxide systems inherently form oxygen vacancies/interstitials upon heating in oxygen-poor/rich gas environments. Such defect reactions must be considered alongside balances of mass action and charge, and exchange of chemical species with the environment. From a detector performance standpoint, control of ion transport by defects introduced by such means is undesirable given susceptibility to changes in thermal history of the solid. As practiced in *semiconductor engineering*, heterovalent impurities are added to the semiconductor host lattice at concentrations above background levels to alter charge balance and introduce temperature-independent concentrations of charged mobile electronic carriers. For example, in the case of wide band gap metal oxides, the incorporation of an acceptor dopant in a binary metal oxide, e.g., A in MO_2_, will lead to the formation of oxygen vacancies according to the following charge balance equation: $\left[{A}_{M}{\prime}\right]=2[{V}_{O}^{..}]$, instead of electronic compensation. Alternatively, a donor dopant might instead introduce an oxygen interstitial, $\left[{D}_{M}^{.}\right]=2[{O}_{i}^{{\prime}{\prime}}]$. Note that Kroger–Vink defect notation is used to describe the net relative electrical charges and lattice positions of the point defect species in the crystal.^[17]^ In this notation ${M}_{S}^{c}$, M corresponds to the atomic species (atoms, vacancy, interstitial), S the lattice site it occupies (e.g., A substitutes on M site, while a vacancy occupies a normally occupied oxygen site), and C corresponds to the electrical charge of the species relative to the site that it occupies (dot—net positive, prime—net negative). The charge of the species is calculated by subtracting the charge on the original site from the charge on the current site. Other examples for different material systems would be, for example, Li interstitial, denoted as ${Li}_{i}^{.}$.

#### Transport

The flux of diffusing ions is determined by variables, such as the packing of the crystalline lattice, the size, charge, and polarizability of both mobile and static lattice ions, as well as the material’s nano- and microstructure. To first approximation, neglecting microstructural effects, the bulk ionic conductivity ${\sigma }_{\text{ion}}$ is defined as

1 $${\sigma }_{\text{ion}}=\left[\text{ion}\right].q.{\mu }_{\text{ion}}={\left[\text{ion}\right].q.\mu }_{ion,0}\text{exp}\left(-\frac{{E}_{m,ion}}{k.T}\right),$$

where $\left[\text{ion}\right]$ is the concentration of mobile ions, ${\mu }_{ion,0}$ is the mobility pre-factor that relates to fundamental crystallographic and material properties, and E_m,ion_ is the migration enthalpy. The ionic mobility ${\upmu }_{\text{ion}}$ (units: m^2^/(V·s)) can be related to the ionic diffusivity *D*_ion,_ (units: m^2^/(s)) according to the Nernst–Einstein relation:

2 $${D}_{\text{ion}}={\upmu }_{\text{ion}}.k.T$$

This definition follows a microscopic picture whereby individual atoms are considered to move through the lattice by hopping *via* adjacent vacant normal or interstitial sites.

The ionic conductivity is typically expected to go through a maximum as a function of defect concentration due to defect interactions.^[18]^ Materials that exhibit optimized ionic conductivities must simultaneously exhibit high concentrations of mobile defects $[\text{ion}]$ and high defect mobilities ${\mu }_{\text{ion}}$.

Open crystal structures with high symmetry (e.g., fluorite and perovskite for oxygen/proton transport, perovskite and garnet for alkali-ion transport) generally offer lower migration enthalpies and more equivalent sites for ion hopping. Additionally, optimized frameworks with tailored diffusion pathways—such as NASICON, LISICON, and LGPS-type structures—have also been identified for alkali-ion transport (Li, Na) through decades of research.^[19]^ Doping to introduce lattice defects is a common strategy to enhance [ion] but comes at times with drawbacks due to either limited dopant solubility or, in the case of highly doped systems (~ > 5–10 atm), defect–dopant association or longer-range defect ordering that contributes to lower ion diffusivity/mobility. In developing radiation detection materials, there is no need to heavily dope systems, as lower dopant concentrations favor higher grain boundary space charge barriers that are beneficial in the optimization of radiation detector performance, as discussed below.

### Grain boundaries and space charge effects

In polycrystalline ceramics, ionic transport is strongly influenced by grain boundaries (GB). The origin of GB resistance in polycrystalline ceramics has been the focus of a large body of work in the field of *Solid-State Ionics*. While secondary phases at grain boundaries can impede ionic transport, grain boundaries often remain resistive to the flow of ions, even in highly purified systems. These boundaries act as barriers to ionic flow due to the presence of space charge regions—zones where charge carriers are depleted or accumulated. These electrostatic barriers develop because they differ in structure, chemistry, and dopant distributions (resulting from segregation phenomena during the high-temperature sintering process), which leads to charge imbalance due to trapped GB core charges. In turn, this causes the mobile point defects to redistribute in the adjacent grains with the following profile:

3 $$\frac{{C}_{defect}\left(x\right)}{{C}_{defect}\left(\infty \right)}=\text{exp}\left(-\frac{z.e.\Delta \Phi (x)}{k.T}\right),$$

where $x$ is the distance from the GB core, $\Delta \Phi (x)$ is the electrical potential relative to the bulk, and z the effective charge of the defect. The spatial distribution of the potential, in turn, depends on the specific details of whether the dopant is mobile under measurement conditions. In a simplified case, most often valid near room temperature, we use a Mott–Schottky model that assumes that only the majority ionic carriers are mobile and can redistribute near the GBs in response to the interface charge, while dopants are kinetically unable to move and have a flat profile up to the grain boundary core. In this case, we can obtain an approximate expression of the grain boundary resistance as a function of grain size (${d}_{g}$) as follows^[20]^:

4 $${R}_{gb}={R}_{bulk}.\left(\frac{\lambda }{{d}_{g}}\right).\left(\frac{\mathit{exp}\left( -\frac{z.e.{\Delta \Phi }_{core}}{k.T}\right)}{\left(\frac{2.z.e.{\Delta \Phi }_{core}}{k.T}\right)}\right),$$

where $\lambda = 2{L}_{D}\sqrt{\frac{\varepsilon {\Delta \Phi }_{core}}{kT}}$ is the space charge width, ${L}_{D}=\sqrt{\frac{\varepsilon kT}{2{e}^{2}{N}_{d}}}$ is the Debye length (where ${N}_{d}$ is the dopant concentration), $\varepsilon$ the dielectric constant, and ${R}_{bulk}=\left(\frac{L}{A}\right){\rho }_{bulk}$ is the bulk resistance which relates to the bulk resistivity ${\rho }_{bulk}$ of the material, defined by Eq. 1, and the electrode geometry. As can be seen in Eq. 4, the grain boundary resistance depends only on three key factors: ${R}_{bulk}$, $\left(\frac{\lambda }{{d}_{g}}\right)$ , and ${\Delta \Phi }_{core}$. Only ${R}_{bulk}$ depends on the electrode geometry, while $\left(\frac{\lambda }{{d}_{g}}\right)$ and ${\Delta \Phi }_{core}$ depend on our choice of material chemistry, sintering protocol, and grain boundary engineering strategy. This has important implications for device design. Assuming equal space charge potential and grain size, from optimal grain boundary engineering and sintering protocols, the ratio of $\frac{{R}_{gb}}{{R}_{bulk}}$ will mainly depend on the space charge potential. By managing to increase the magnitude of the space charge potential, one can therefore design devices with extremely low dark currents, desirable for the development of radiation detection technologies.

## Theory: Optoionic effects

### Radiation response

**Figure 2:**
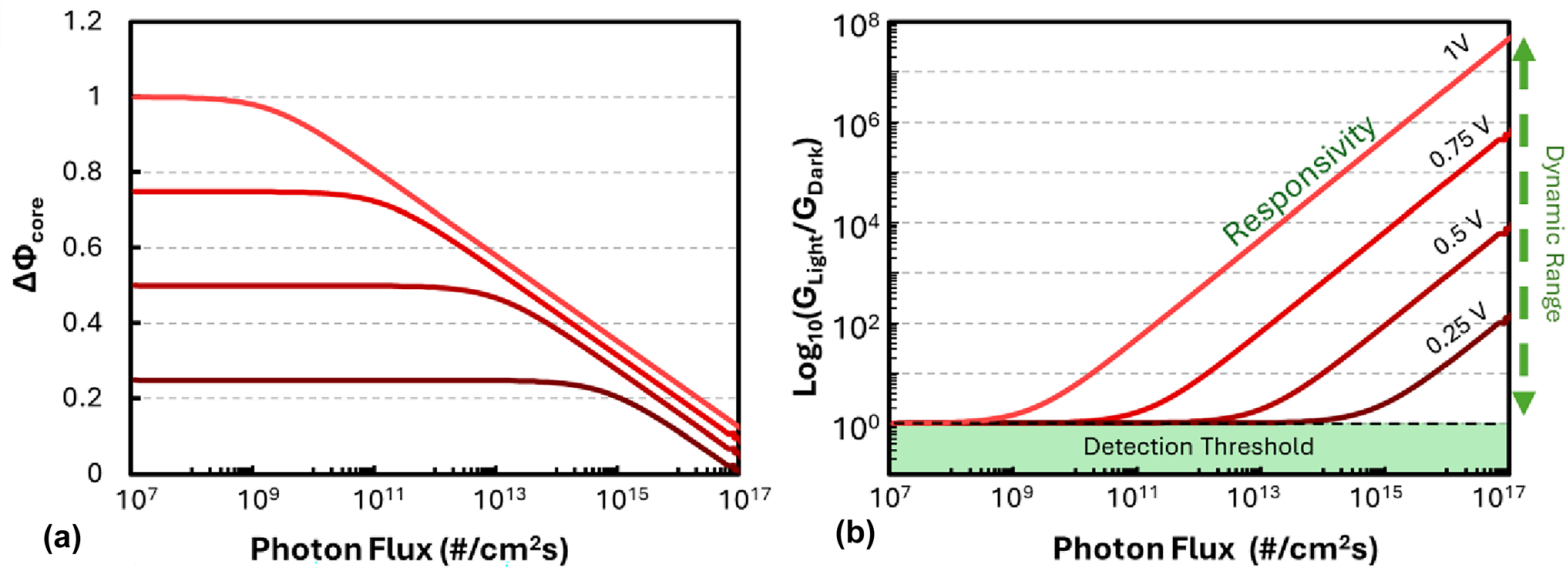
(a) The dependence of the space charge potential as a function of the logarithm of photon flux (#/cm^2^s)—reproduced from Ref. 15 (Defferriere et al., Nature Materials 21, 438–444 (2022). Copyright 2022 by Springer Nature)—plots calculated for 3at% Gd-doped CeO_2_ at 300℃, (b) Logarithm of expected conductance ratio as a function of photon flux (/cm^2^s) depending on the dark space charge potential, as described by Eq. 5, obtained by replotting (a).

The theoretical foundation of the grain boundary optoionic effect lies in the interplay between grain boundary space charge fields and photogenerated charge carrier trapping dynamics. The space charge potential (${\Delta \Phi }_{core}$) is a critical parameter that governs grain boundary resistance as derived previously. The physics of charge carrier splitting and trapping at space charge-controlled grain boundaries in semiconductor materials was derived in the 1980s by Seager et al.^[21]^ An expression describing the relationship between the grain boundary space charge potential with the absorbed photon flux was derived through a detailed kinetic model accounting for key materials parameters such as the minority carrier diffusion length, hole/electron capture cross section at the grain boundary, concentration of empty trap states in the grain boundary core, and the fraction of over-barrier majority carrier current captured at the grain boundary that depends on position of Fermi level.^[21]^ Importantly, this equation does not consider the distance between electrodes as it is focused solely on describing charge trapping kinetics at the grain boundaries. The equation does not possess an exact solution but can be solved numerically (as we previously reported for our model material Gd-doped CeO_2_ in Ref. 15), with the general functional dependence for different dark space charge potentials illustrated in Fig. 2(a). One notices that when the initial dark grain boundary space charge potential barrier is higher, the absorbed photon flux required to start reducing the potential barrier is smaller by orders of magnitude. This has important implications for designing radiation detectors as it implies higher optoionic sensitivities for grain boundaries with larger dark space charge potential barriers.

To better understand how Fig. 2(a) relates to radiation detection device performance, we can define two different conditions, ${R}_{gb,Dark}$ and ${R}_{gb,Light}$, based on Eq. 4. Noting that ${R}_{bulk}$ and ${d}_{g}$ are expected to be independent of light intensity, then the only difference is the space charge potential value under illumination conditions. By considering the ratio $\frac{{R}_{gb,Dark}}{{R}_{gb,Light}}$, we obtain the following relationship for the change in resistance as a function of light intensity:

5 $$\frac{{R}_{gb,Dark}}{{R}_{gb,Light}}=\frac{{G}_{Light}}{{G}_{Dark}}=\sqrt{\frac{{\Delta \Phi }_{core,Light}}{{\Delta \Phi }_{core,dark}}} \mathit{exp}\left( -\frac{2.e.\left(\Delta {\Phi }_{core,dark}-\Delta {\Phi }_{core,light}\right)}{k.T}\right)$$

As displayed in Fig. 2(b), where we display the functional dependence of Eq. 5, the magnitude of the space charge potential controls exponentially the ratio of the resistance modulation window ($i.e \frac{{G}_{Light}}{{G}_{Dark}}$)—which can be related, in turn, to the radiation detector *dynamic range*. Quadrupling the space charge potential from 0.25 to 1 V is expected to result in an increase of the dynamic range on the order of ~ 10^6^ for equal radiation dose. Moreover, the optical sensitivity, which relates to both the *detection threshold* (the minimum radiation dose required to generate a measurable signal) and *responsivity* (the rate of change in detector output per unit increase in radiation dose, measured as the slope of the curve in Fig. 2(b)), is also both connected to the magnitude of the space charge potential in the dark. Following the previous example, quadrupling the space charge potential can lead to an improvement of a factor 10^6^ in the minimum photon flux needed to achieve a detectable signal (from 10^15^ to 10^9^ #.cm^−2^.s^−1^). This indicates that the radiation response can be engineered by simply increasing the grain boundary space charge potential, or equivalently by increasing the dark resistivity. In the Mott Schottky model, the space charge potential can be related to the net core charge ${Q}_{core}$, the dopant concentration $[Dopant]$, and the dielectric constant $\varepsilon$ according to

6 $${\Delta \Phi }_{core}= -\frac{{Q}_{core}^{2}}{8.e. \varepsilon .[Dopant]}$$

Equation (6) highlights the importance of control of ${Q}_{core}$, given its quadratic relationship to the space charge potential ${\Delta \Phi }_{core}$. In future reports, we will show explicitly how changing the number of trapped charges at the grain boundary allows one to systematically tune the materials’ radiation sensitivity.

Moreover, we note in Eq. 5 that electrode geometry does not play a role in the expected response. This insensitivity to electrode geometry can be rationalized by the fact that the grain boundaries act as *virtual electrodes*. This is in stark contrast to traditional semiconductor detectors where the distance between electrodes and, therefore, the mobility-lifetime product ($\mu .\tau ),$ plays an essential role in defining the collection efficiency of the device. In our material, the photogenerated charge carriers need only diffuse to the grain boundary space charge region, at which point the space charge field takes care of splitting them efficiently. Following modern manufacturing processes, achieving polycrystalline solid electrolytes with nanocrystalline grain sizes on the order of 10’s nm is feasible, thereby ensuring extremely short transport lengths. Consequently, one can engineer devices with different bulk interaction volumes, all the while maintaining the same optoionic responsivity. This provides flexibility for device design based on measurement requirements (i.e., expected background signals vs absolute conductance) and measurement needs (desired interaction volumes). For example, devices with very large volumes could be engineered where maximized ionizing radiation stopping power is required or, alternatively, extremely small volumes, where extremely fine pixelated arrays are desired. This contrasts with existing semiconductor technologies where large volumes can compromise collection efficiency and downsizing pixel size can result in decreasing quantum efficiency and increased electronic cross- talk. It is further worth noting the general applicability of Eq. 5 to other material systems with similar grain boundary properties but different ionic mobilities, that would exhibit similar responsivities, but far different response rates, as discussed in the following.

### Dependence of response rate on material properties

The design of practical radiation devices covers a broad range of device applications, from simple dosimetry, where absorbed radiation dose is measured—with detectors providing stable, linear response over broad dose ranges, to more advanced spectroscopic applications that require high collection efficiency and rapid transit times to distinguish closely timed events and enable high energy resolution for accurate identification of specific radiation energies. While high sensitivity is a necessary condition for developing a radiation detector, it is an insufficient requirement for high performance. Transit times are moreover critical and depend on the response rate of the optoionic phenomena. This rate is governed by the dynamics of the photogenerated carriers and their interaction with grain boundaries alongside the distribution dynamics of the ionic defects in response to the change in space charge properties. The key factors influencing the response rate are as follows: 1. Electronic charge generation, 2. transport to the space charge region, 3. charge trapping/detrapping, and 4. carrier lifetime, v. rate of ionic diffusion. Ultimately, the slowest steps will control the optoionic response rate.

**Figure 3:**
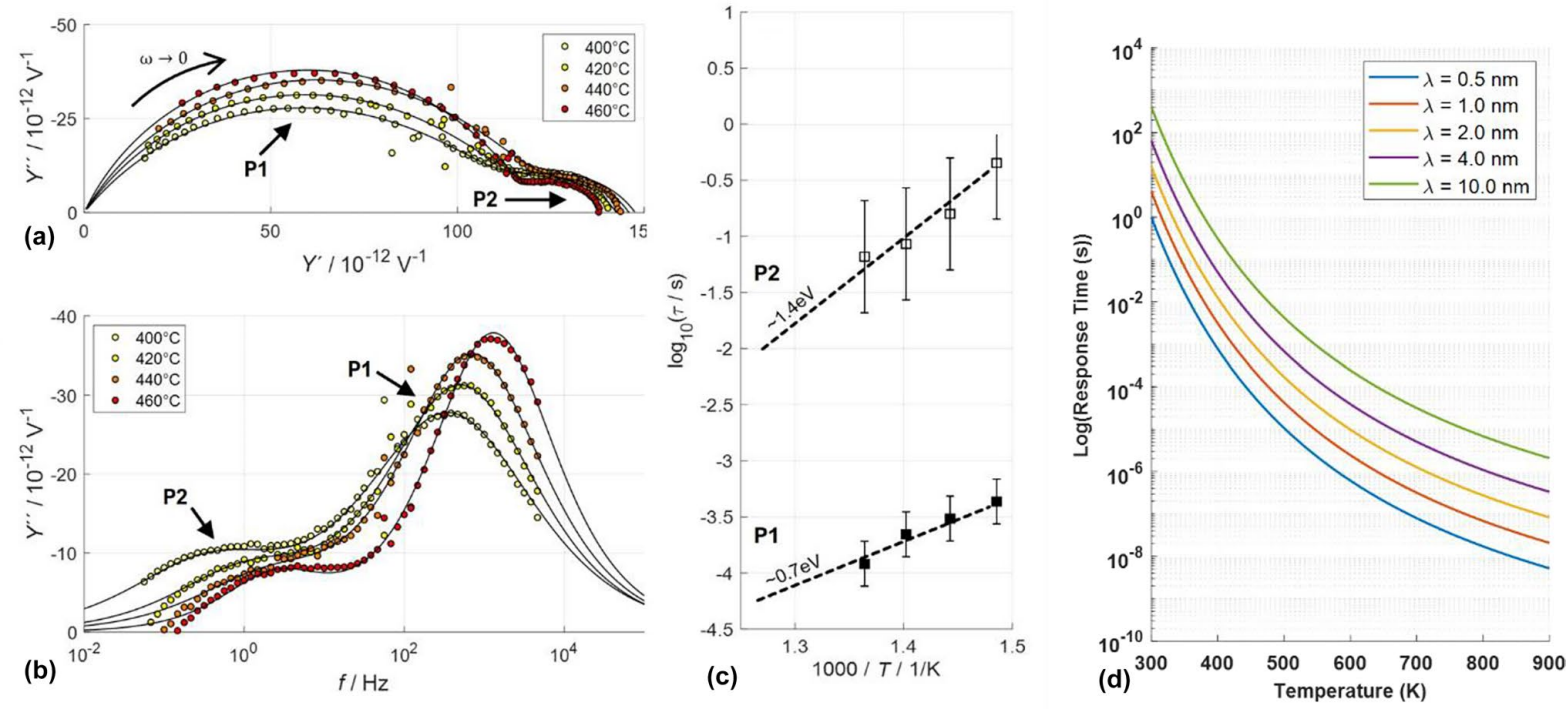
Frequency-dependent optical modulation of resistance response by IMPS. a–d, IMPS spectra measured on polycrystalline 3 at% Gd-doped CeO_2_ in synthetic air. Nyquist plots (a) of photocurrent admittance (*Y*, consisting of real, *Y*’, and imaginary parts, *Y*’’) and (b) plots of imaginary components of *Y*(*ω*) as a function of frequency. Wavelength of light source: 375 nm. The points represent the measurements, while solid lines are model fits using an empirical equivalent circuit model after correcting for an inductive artifact caused by the measurement setup. (c) Arrhenius plots of obtained time constants for the individual processes, with the dominant process P1 possessing activation energy and time constant consistent with ${\text{D}}_{{\text{O}}^{2-}}$. Reprinted from Ref. 15 (Defferriere et al., Nature Materials 21, 438–444 (2022). Copyright 2022 by Springer Nature). (d) Modeled response time using Eq. 7 related to diffusion process for oxygen ions in 10 at% Gd-doped CeO2 as a function of temperature depending on a fixed space charge width (λ), where the diffusion coefficient is calculated from conductivity and defect concentration in Ref. 22.

Previously, we measured the dynamics of the optoionic response in 3 at% Gd-doped CeO_2_ thin films devices by intensity-modulated photocurrent spectroscopy (IMPS)^[15]^—as illustrated in Fig. 3(a, b, c). This allowed us to deconvolute the optoionic response in the frequency domain as a function of temperature and identify the rate-limiting process. The rate of change of space charge resistance was found to be governed by the out-diffusion of oxygen ions from the space charge regions in response to the reduction in space charge field magnitude. To a first-order approximation, the diffusion process could be described by Fick’s laws of diffusion, as it relates to a simple internal redistribution of defects upon illumination-induced lowering of the electric field, where the time ($\tau$) required for oxygen vacancies to redistribute is given by

7 $$\tau =\frac{{\lambda }^{2}}{{2.D}_{ion, bulk}}.$$

Considering the resistance modulation mechanism depends on the redistribution kinetics of ions out of the space charge region back into the bulk, it is therefore related to the diffusion dynamics of the ions in the bulk (where ${D}_{ion,bulk}=\frac{{\left[defect\right]}_{bulk}}{[lattice site]}{D}_{defect})$ which is opposite to traditional macroscopic ion diffusion processes, that depend on the diffusion of ions across the carrier-depleted space charge layer at grain boundaries. This implies that the response time is not expected to be affected by the magnitude of the depletion of defects in the space charge region. This contrasts with the RC time constant of an AC measurement, which depends on the instantaneous values of resistance and capacitance, the former of which depends on the magnitude of the depletion of carriers in the space charge zone. As we are simply measuring changes in total resistance due to local changes in carrier concentration, the RC time constant does not directly dictate the rate of this redistribution.

**Figure 4:**
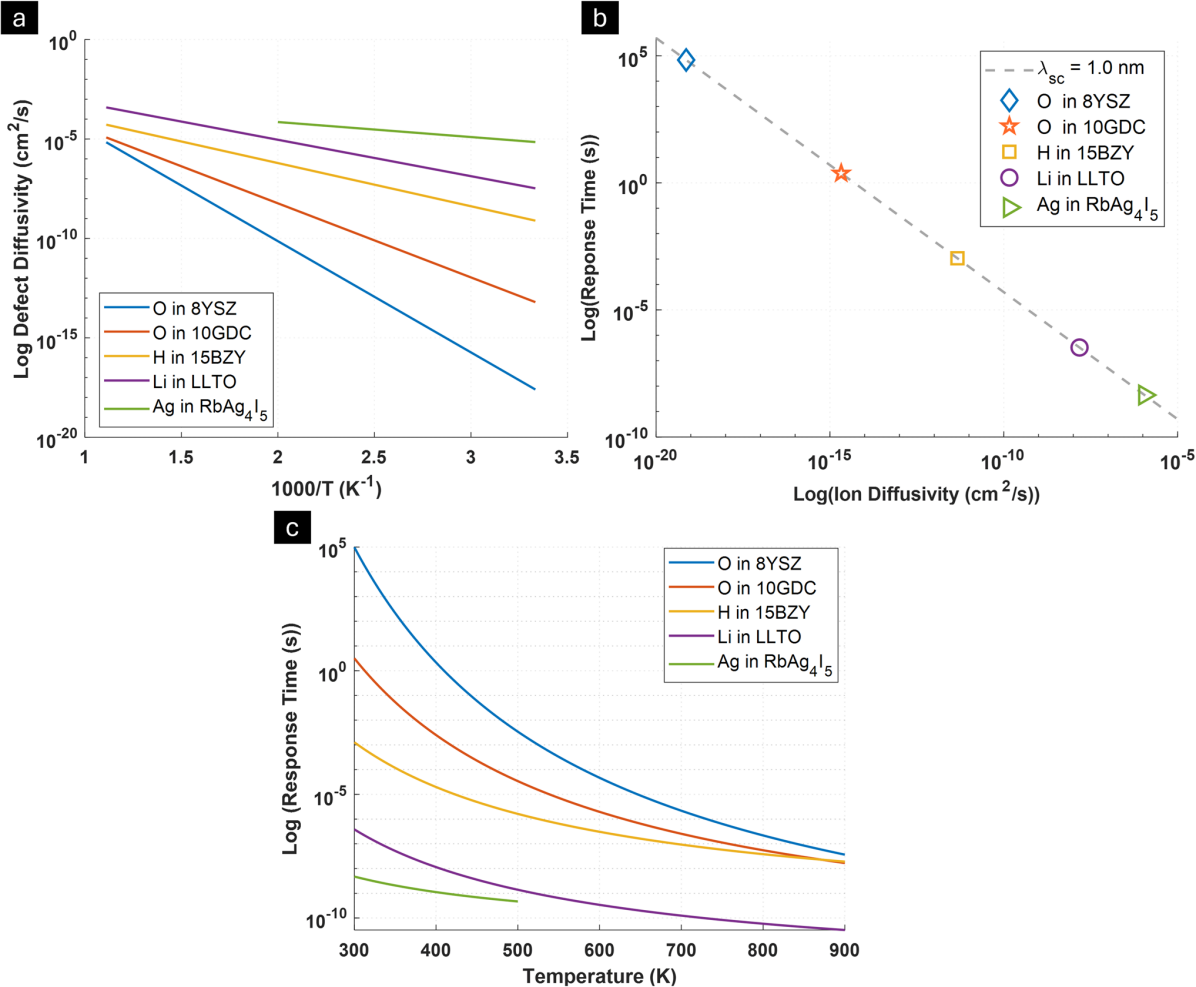
(a) Arrhenius plots representing log_10_ defect diffusivity vs. 1000/T for different ionic defect types in model fast ionic conductors (oxygen ions in 10 at% Gd-doped CeO_2_ and 8 at% Y-doped ZrO_2_, OH^+^ in 15 at% Y-doped BaZrO_3_, Li ions in Li_0.3_La_0.57_TiO_3_, Ag ions in RbAg_4_I_5_) calculated from Refs. 20, 22–25 and extrapolated to high temperatures (note that RbAg_4_I_5_ is expected to melt above 228°C). (b) Log–Log plot of calculated response time (s) vs Ion Diffusivities for arbitrary 1 nm space charge width at 30℃ (calculated assuming ideal $\frac{\left[\text{defect}\right]}{[\text{lattice site}]}$ for all compounds), (c) Expected variation of Log_10_ (response time (s)) vs temperature (in Kelvin) for different compounds.

As shown in Eq. 7, and displayed in Fig. 3(d), the response time is dependent on both the space charge width and temperature. While a higher dark space charge potential is expected to increase the response time, due to a larger space charge width, which is undesirable, this is expected to only follow a linear dependence (as τ ∝ λ^2^ ∝ $\Delta {\Phi }_{core,dark}$) which is significantly outweighed by the exponential gain in optoionic sensitivity (Eq. 5), indicating a favorable trade-off for enhancing detector performance. Moreover, temperature is an effective approach at controlling the response time as the ion mobility can tuned over orders of magnitude due to the thermally activated nature of ionic transport, as outlined in Eqs. 1 and 2. This can be used to offset any modest increases in response time due to higher space charge potential. Additionally, different ionic species in different solids possess dramatically different activation energies and, therefore, can achieve exponentially different diffusivities as a function of temperature, as displayed in Fig. 4(a). Some compounds, such as the oxygen ion conductors, will be expected to exhibit low diffusivities near room temperature (diffusivities on order of 10^–14^–10^–18^ cm^2^/s translating to response times on order of seconds to 1000’s of seconds), but become much more rapid at higher temperatures where they excel in stability. Lithium, proton, or silver conductors, on the other hand, can be expected to have much faster response times near room temperature (diffusivities ~ 10^–6^– 10^–11^ cm^2^/s, translating to response times on the order of nano to microseconds)—as shown in Fig. 4(b, c). In future reports, we will show how operating detectors at different temperatures, or by selecting different material systems with higher bulk ionic diffusivities, allow one to access a broad range of radiation response rates.

## Future directions and conclusions

Grain boundary-based optoionics offers a transformative approach to radiation detection, leveraging the unique interaction of light and radiation with ionic conductors to create scalable, robust, and cost-effective devices. By addressing the limitations of semiconductor-based detectors, optoionic systems promise to revolutionize applications in nuclear energy, security, and environmental monitoring. While the principles discussed so far highlight the potential of such materials to be used as radiation detectors, demonstration of such feasibility is still underway.

So far studies have been performed on oxygen ion conductors Gd-doped CeO_2_ under various sources of irradiation (UV and Gamma Ray), both in thin film^[15]^ and bulk formats,^[16]^ resulting in resistance normalized sensitivities ($\frac{\frac{{G}_{Light}}{{G}_{Dark}}}{\left[e-h generated\right]})$ on the order of 10^–15^–10^–13^ cm^3^.s (based on an incident flux and integrated over device volume). Comparing this against commercial Si photodiodes,^[26]^ where dark resistances can be on the order of GΩ, we can convert sensor noise floor levels to equivalent normalized sensitivities, that is on the order of 10^–7^ cm^3^.s. In this example, if one wishes to compete with state-of-the-art radiation detectors, sensitivity enhancements of ~ 10^7^ would be necessary. While such enhancements may seem large, ionic materials studied to date were not pre-engineered, with grain boundary space charge potentials on the order of ~ 0.2 V. As described above, grain boundary space charge engineering should allow exponential enhancements in optoionic sensitivity. Simply tripling or quadrupling the space charge potential is expected to result in sensitivity enhancements by factors of 10^4^–10^6^. Successful grain boundary engineering will depend on the ability to accurately control the amount of trapped core charges at the grain boundaries. Moreover, developing proper manufacturing methods that can be applied to both thin film technologies and bulk ceramics, allowing for homogeneous modification of grain boundary properties, will be vital for enabling high energy resolution applications, such as spectroscopy. This stems from the fact that variability in interface properties between different grain boundary regions will negatively impact the quality of radiation-induced signals. Since conventionally fabricated polycrystalline ceramics typically contain a range of grain sizes, misorientation angles between grains and variations in concentrations of impurities segregated to the grain boundaries, this will lead to degraded response. Advanced doping strategies and carefully optimized sintering protocols are thus required to achieve uniform grain sizes with post-sintering processes to homogenize dopant profiles. An improved understanding of the relationship between space charge physics, grain boundary structure, and grain boundary chemistry will furthermore be required to develop more advanced models that can be used in accurately predicting the radiation detection performance of materials.

As discussed above, detectors for use in advanced pulse counting spectroscopic applications require operation on fast timescales (μs). To date, the oxygen ion conductors have demonstrated response rates on the order of seconds near room temperature owing to low oxygen ion diffusion coefficients. At higher temperature, milliseconds rates have been achieved, consistent with the exponential dependence of diffusivity on temperature. Moving to different ionic conductors with much higher ionic mobilities (Li, H or Ag) is expected to result in even faster response times.

The development of radiation detectors will also depend on our ability to create fully functional devices. While the physics of signal generation in novel ionic-based radiation detectors was outlined, additional considerations will be needed to achieve stable response, including appropriate electrodes capable of minimizing polarization. Often considered a challenge for semiconductor-based devices where ionic species are mobile (e.g., TlBr, MAPI, CsPbrBr_3_), ionic polarization associated with the pile-up of ionic species at the electrode interfaces under applied potential results in device signal shifts due to interference with the charge collection process at the electrode interfaces.^[27,28]^ Over extended periods of time, it can even lead to material instability and degradation due to irreversible chemical reactions.^[29,30]^ This process is expected to be even more significant in optoionic detectors as ion conduction dominates over electronic conduction. While the charge collection process across the electrode is not of relevance for our device design, the pile-up of ionic species will nevertheless create an internal chemical potential gradient that opposes externally applied DC voltages and causes the ionic flux to tend toward zero. AC measurements with ion-blocking electrodes could present an interesting operational mode to prevent this polarization phenomenon. However, the inherently high grain boundary capacitance (~ nF) can be expected to limit sampling frequencies (potentially to the Hz range or lower), posing challenges for high-rate applications like spectroscopy and introducing potential asynchronous sampling biases. Alternatively, we note that similarities with solid-state battery design can be drawn, whereby only ionic carriers are expected to travel between two ionically reversible electrodes, enabling a stable ionic flux. This can be achieved if the electrodes are ionically reversible and electronically conductive. Mixed ionic and electronic conducting electrodes, which exhibit both high ionic and electronic conductivity, while also allowing for large changes in chemical capacities associated with easy insertion and removal of ionic carriers, can be employed for this purpose. Charge transport kinetics of ions and electrons in these materials can, at times, depending on the type of ions being transferred, differ depending on operating temperature, and therefore identifying the right mixed conducting materials and engineering their transport properties for desired operating conditions will be critical. Future efforts should therefore be directed toward understanding how charge transfer kinetics across the electrodes influence device response and how these transfer processes can be altered by incident radiation**.**

## Data Availability

Raw data directly obtained from Refs. 20,22–25. Additional data that support the findings of this study are available from the corresponding authors upon reasonable request.
